# Clinical Modalities for Enhancing Reproductive Efficiency in Buffaloes: A Review and Practical Aspects for Veterinary Practitioners

**DOI:** 10.3390/ani14182642

**Published:** 2024-09-11

**Authors:** Stefan Coman, Daniel Ionut Berean, Raluca Cimpean, Simona Ciupe, Ioan Coman, Liviu Marian Bogdan

**Affiliations:** 1 Department of Reproduction, Faculty of Veterinary Medicine, University of Agricultural Sciences and Veterinary Medicine Cluj-Napoca, Calea Manastur 3-5, 400372 Cluj-Napoca, Romania; coman.stefan25@yahoo.com (S.C.); simona.ciupe@usamvcluj.ro (S.C.); liviu.bogdan@usamvcluj.ro (L.M.B.); 2 Department of Animal Breeding and Food Safety, Faculty of Veterinary Medicine, University of Agricultural Sciences and Veterinary Medicine Cluj-Napoca, Calea Manastur 3-5, 400372 Cluj-Napoca, Romania; calina-raluca.cimpean@student.usamvcluj.ro; 3 DSVSA Brasov, 500483 Brașov, Romania; ioan_coman63@yahoo.com

**Keywords:** buffalo reproduction, estrus synchronization, ovulation induction, reproductive pathologies

## Abstract

**Simple Summary:**

This review considers two key areas of reproductive management in buffaloes: assisted reproductive technology (ART) techniques, including estrous synchronization (ES) and artificial insemination (AI), and clinical approaches for functional and inflammatory reproductive diseases. It covers methods to induce ovulation and corpus luteum formation in buffaloes, and addresses prevalent reproductive pathologies like retained placenta, metritis, anestrus, and repeat breeding. This review highlights the importance of effective reproductive management strategies to enhance fertility and overall herd productivity in buffalo farming.

**Abstract:**

This review aimed to bring a comprehensive analysis of key clinical strategies for enhancing reproductive efficiency in buffaloes, a species that exhibit low reproductive performance under conventional reproductive management compared to that exhibited by cattle. It considers key ART techniques including estrus synchronization for artificial insemination, and ovulation induction, highlighting their role in improving fertility and overall herd productivity. However, it also addresses common postpartum inflammatory and functional reproductive disorders, discussing their diagnosis and treatment protocols, stressing their impact on the overall reproductive outcome in buffalo farming.

## 1. Introduction

Enhancing reproduction in buffalo holds significant importance in sustainable agriculture and the dairy industry. With the demand for buffalo milk products rising globally, optimizing reproductive efficiency becomes crucial. Buffalo production plays a critical role in the global food production chain, particularly in Asia, where these animals are a cornerstone of the agricultural economy. Buffaloes contribute significantly to meat, milk, and leather production, with their milk being a staple in many countries, notably India and Pakistan, which together produce the majority of the world’s buffalo milk. This species is highly valued for its adaptability to diverse environments, including tropical and subtropical regions, where other livestock may struggle. Economically, buffaloes are crucial for smallholder farmers, providing a source of income, food security, and employment. The global buffalo population is growing, reflecting the increasing demand for their products in both local and international markets. This growth underscores the importance of buffaloes in sustainable agriculture and rural development, particularly in regions where they serve as a primary asset in both subsistence and commercial farming [1]. Various techniques have been developed to improve reproduction in buffalo, ranging from genetic selection and assisted reproductive technologies (ARTs) to nutritional management and reproductive health monitoring. By implementing these strategies, farmers can boost fertility rates, increase milk production, and ensure the long-term sustainability of buffalo farming [2].

Buffaloes have traditionally been prized by small-scale farmers for their robustness in hot climates and resilience to diseases and stress, primarily serving as sources of draft power [3]. However, deliberate efforts have been made over the years to transition these animals from draft work to dairy production through genetic improvement programs, leading to the establishment of buffalo-based enterprises. The socioeconomic importance of buffalo production has increasingly captured the attention of farmers, as it plays a crucial role in advancing the livestock industry in many developing nations [1]. However, despite the critical role buffaloes play in these economies, their reproduction presents considerable challenges. These challenges stem from a complex interplay of intrinsic factors, such as genetic and physiological limitations, as well as extrinsic factors, including environmental conditions, management practices, and resource availability [2]. Compared to cattle, buffaloes exhibit lower reproductive efficiency due to various factors, including inadequate estrus manifestation, silent heat periods, pronounced seasonal infertility, extended postpartum anestrus, prolonged calving intervals, delayed onset of puberty, etc. [3,4,5]. ARTs are crucial for improving reproductive efficiency in buffaloes. Among these, fixed-time artificial insemination (FTAI) is the most widely used technique, designed to synchronize ovulation within a herd to facilitate timed insemination [6,7,8]. FTAI does not aim to optimize the utilization of ovarian follicular reserves but rather focuses on grouping functional ovulations for practical breeding management. Other techniques, such as in vitro embryo production, intracytoplasmic sperm injection, oocyte cryopreservation, and somatic cell nuclear transfer, are less commonly used in buffalo practice, even in advanced cattle operations, due to their high costs and limited economic benefits [9,10,11].

Estrus synchronization (ES) is vital for enhancing reproductive efficiency in buffalo herds, allowing farmers to optimize AI programs to enhance genetic progress. Challenges such as insufficient estrus manifestation and prolonged intervals from calving to estrus, especially during hot seasons when fertility declines, hinder reproductive efficacy [12]. To address these issues, various protocols have been developed to synchronize estrus and ovulation by manipulating the corpus luteum (CL), often using prostaglandins or progesterone. While short-term progesterone protocols typically do not extend the luteal phase, recent studies indicate the need for more precise control of follicular development to improve ovulation synchrony and fertility outcomes. This has led to the exploration of hormone regimens involving gonadotropin-releasing hormone (GnRH), follicle-stimulating hormone (FSH), luteinizing hormone (LH), equine chorionic gonadotropin (eCG), human chorionic gonadotropin (hCG), prostaglandins, progesterone, and estradiol [13].

The LH is integral to the ovulation process, making knowledge of the interval between the LH surge and ovulation crucial for accurately timing artificial insemination in buffaloes. Despite various studies on hormonal fluctuations and circulating LH levels, information on the specific timeframe between the LH surge and ovulation is limited [14,15,16]. Common approaches to induce ovulation include using GnRH analogs, which stimulate LH release, and hCG, which mimics LH’s effects. Additionally, optimizing nutrition and minimizing stress can support natural ovulation by maintaining hormonal balance and reproductive health [17,18]. 

Reproductive pathologies in buffaloes encompass a range of disorders affecting the reproductive system, which can influence fertility and breeding success. Some common reproductive pathologies described in buffaloes include the following: endometritis, metritis, cystic ovarian disease, retained placenta, pyometra, anestrus, dystocia, neoplasia, uterine prolapse, and ovarian hypofunction [19,20,21]. Proper diagnosis and treatment management of these reproductive pathologies are essential for maintaining herd fertility and optimizing breeding programs in buffalo populations. Veterinary intervention, including thorough clinical examination, diagnostic imaging, hormonal assays, and targeted treatment protocols, is crucial for addressing reproductive disorders and promoting reproductive health in buffaloes [22,23].

**Figure 1:**
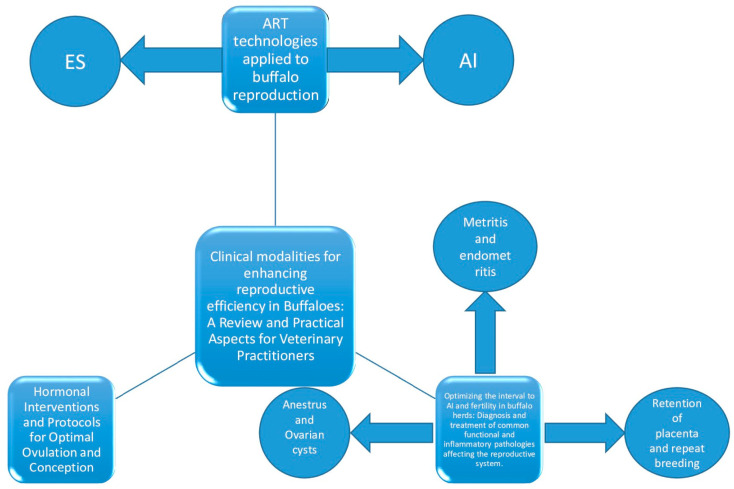
Diagram of clinical approaches in buffalo reproduction. The clinical approach to buffalo reproduction allows for the management of reproduction from three perspectives: control and induction of ovulation, ART techniques such as ES and AI, and control of major reproductive pathologies.

The present review aims to analyze the clinical modalities through which the reproductive activity of buffaloes can be positively influenced and to provide practical solutions to veterinary practitioners. The review primarily focuses on ART techniques applied to buffaloes, such as estrus synchronization and the stimulation of ovulation and corpus luteum formation through various hormones. Additionally, it addresses the diagnosis and treatment of reproductive pathologies that significantly impact buffalo production (Figure 1).

## 2. ART Technologies Applied to Buffalo Reproduction

The primary focus of reproductive biotechnologies in buffaloes should be on those ARTs that have practical applicability and are consistent with advancements in buffalo reproductive management. Among these, AI remains the most widely implemented due to its effectiveness and ease of use [24,25]. The success of AI has been bolstered by a deeper understanding of buffalo reproductive physiology, improvements in semen cryopreservation techniques, and the development of various ES protocols. These developments have expanded the scope of AI implementation in buffalo breeding programs, with ES protocols demonstrating promising outcomes when applied in practice. The discussion will focus on the ARTs that have shown the most promise and practicality in improving reproductive outcomes in buffalo populations [26,27,28].

ES combined with FTAI addresses two key challenges in buffalo breeding: suboptimal overt estrus expression and breeding seasonality. Research in cattle indicates that successful ES in buffaloes also relies on having a functionally competent dominant follicle (DF) at the time of ovulation [29]. While corpora lutea (CL) are crucial for PGF-based protocols, they are not necessary for progesterone-based protocols. In buffaloes, ES involves using pharmaceutical agents such as prostaglandins, progestins, and GnRH, either alone or in combination. It is important to note that GnRH is specifically used to induce ovulation and should not be employed for ES itself. Similarly, progesterone and GnRH do not influence luteal regression in the context of ES protocols. Instead, these agents regulate CL regression, follicle growth and regression, and ovulation. Research underscores the critical roles of the DF and CL in the follicular dynamics that underlie ES protocols. Understanding these dynamics is essential for effective ES and FTAI in buffaloes. Specifically, ES operates either through the progression of the DF to ovulation following prostaglandin-induced luteolysis or through the regression of the DF, followed by the development of a new DF that matures to ovulation, as seen in progestin- and GnRH-based protocols [30].

### 2.1. ES Protocols for Buffaloes

#### 2.1.1. Ovsynch Protocol

The Ovsynch protocol (Table 1) is a widely utilized ovulation synchronization method in cyclic buffaloes, consisting of two injections of GnRH and a single administration of prostaglandin F2 alpha (PGF2α). This protocol is designed to synchronize ovulation and facilitate timed artificial insemination (TAI). The initial GnRH injection induces the ovulation of the existing DF and promotes the formation of a new follicular wave, which will become the ovulatory wave. This is typically followed by the administration of PGF2α 7 days later to induce luteolysis of the existing CL. A second GnRH injection is administered 48 h after PGF2α to trigger ovulation of the newly developed dominant follicle, allowing for precise timing of AI 16 to 24 h post-GnRH [31,32].

Additional factors affecting buffalo response to this protocol need to be considered, including the buffalo’s response to ovulation after the first GnRH, influenced by follicular dynamics, the presence of the CL, and the efficiency of luteolysis after PGF2α administration. The diameter of the follicle at the time of the second GnRH injection and its subsequent response are also critical factors. The interval to ovulation after the second GnRH injection needs to be described, as it directly influences the success of the protocol. In the summer season, after the Ovsynch protocol, ovulation was observed in 60% of acyclic buffaloes and 81% of cyclic buffaloes, indicating a higher ovulation rate in cyclic buffaloes compared to their non-cycling counterparts during this period. These observations highlight the importance of updating our understanding of the factors that affect buffalo response to the Ovsynch protocol [33].

Ovulation of the largest follicle at the time of the first GnRH administration in buffaloes is influenced by the size of the dominant follicle. When the largest follicle was greater than 8 mm, ovulation occurred in 87.5% of heifers and 100% of adult buffaloes [34]. The ovulation response also depends on the follicle’s stage, with 75% ovulation during the growth phase and only 16.67% during the regression phase of the first wave. The first anovulatory wave in buffaloes tends to reach maximum size earlier and remains in a static phase longer, indicating an earlier loss of LH receptors compared to cows. A new follicular wave starts a day after the first GnRH dose, leading to the development of a new dominant follicle with a diameter of 1.03 cm by day 7 after treatment [29]. The interval between the first GnRH administration and the onset of a new follicular wave is 48 to 54 h, with luteolysis induced by PG administration seven days later allowing the new or continued growing follicle to reach ovulatory size, thereby inducing estrus. The second GnRH treatment ensures ovulation. Ovulation following the first GnRH injection is a useful preliminary screening tool for selecting buffaloes for insemination, as those that ovulate have a significantly higher pregnancy rate (81%) compared to those that do not (22.3%). The Ovsynch protocol has been used widely with estrus rates varying from 41.6% to 91.9% and conception rates from 11.11% to 68.8%. Estrus synchronization with Ovsynch in buffalo heifers has yielded lower conception rates (18.8–40%) compared to adult buffaloes [34], with higher estrus and conception rates during the breeding season (87.5% vs. 36.3% and 40% vs. 11.1%) [29].

Several variations in the Ovsynch protocol have been developed recently, presenting feasible options for improving reproductive indices. Insulin (Table 1) has been explored in the management of anestrus in buffaloes because of its role in modulating the terminal follicle development together with insulin-like growth factors and the gonadotropins. Insulin indirectly influences reproductive processes by promoting hepatic secretion of insulin-like growth factor I (IGF-I), which in turn may enhance follicular competence for estrus and ovulation in association with gonadotropin. This rationale sustains the use of insulin in modified Ovsynch protocols, which have shown promising pregnancy rates [35,36]. In modified Ovsynch protocols for buffaloes, insulin is administered subcutaneously at a dose of 0.1 to 0.2 IU/kg body weight on days 0 and 7, alongside GnRH and PGF₂α, to improve follicular development and synchronization outcomes [36]. In a study by Gupta K.K. et al. (2015) [31], the impact of insulin administration within Ovsynch protocols was investigated, revealing that conception rates following timed artificial insemination (TAI) were significantly higher in modified Ovsynch protocols incorporating insulin or a double dose of GnRH (40%) compared to the standard Ovsynch protocol (20%) in anestrous buffaloes. The observed conception rates (20–40%) were consistent with other studies utilizing the GnRH-PGF2α-GnRH (G-P-G) protocol, which reported low conception rates due to factors such as early ovulation and sub-functional CL. The presence of larger follicles at the start of the Ovsynch protocol is crucial because they are more likely to respond effectively to hormonal stimulation, leading to successful ovulation. This enhances the synchronization process and improves conception rates by ensuring that the follicles are sufficiently developed to ovulate in response to the GnRH and PGF₂α treatments. In non-cyclic buffaloes, ovulation occurs over a more extended period (12–36 h, with an average of 26 ± 4.8 h), which may contribute to lower conception rates, particularly during the summer season. The use of insulin in these modified protocols has shown potential to improve conception rates, similar to findings in cattle [37,38]. Notably, the highest conception rates in postpartum buffaloes (66.66%) were achieved using an insulin-modified Ovsynch protocol, where the second GnRH dose was entirely replaced with insulin administered on days 8, 9, or 10 [36]. This suggests that insulin can be a valuable component of Ovsynch protocols, particularly in managing postpartum anestrus in buffaloes.

The administration of a dose of PGF2α 12 days before initiating the protocol has not shown any improvement. However, according to the same author, the implementation of a double Ovsynch protocol has enhanced both the ovulation rate (88.3%) and conception rate (44.4%) [38]. In another study, the authors modified the Ovsynch protocol by injecting the females with the first GnRH (gonadotropin-releasing hormone, 400 μg) on day 0, PGF2α (prostaglandin F2α, 0.5 mg) on day 7, and the second GnRH along with mifepristone (0.4 mg/kg) simultaneously on day 9. Additionally, hCG (human chorionic gonadotropin, 2000 IU) was administered on day 5 after AI. The modified protocol, in comparison to the classic Ovsynch protocol, improves estrus response (86.1% vs. 75.8%), ovulation (81.9% vs. 65.2%), and pregnancy rates (45.4% vs. 35.8%) in crossbred multiparous buffaloes during the peak breeding seasons [39,40]. Other studies have also reported the beneficial impact of the mifepristone administration in combination with the Ovsynch protocol. Buffaloes treated with a dose of 0.4 mg/kg mifepristone combined with the Ovsynch protocol exhibited significantly higher (*p* < 0.05) estrous response (82.4%), ovulation (94.1%), and pregnancy rates (47.1%) (Table 1). The injection of mifepristone along with the second GnRH injection in buffaloes significantly improved (*p* < 0.05) the pregnancy rate (35.3%) compared to its administration before or after the second GnRH of the GPG protocol [40]. These modifications, particularly the incorporation of mifepristone and the double Ovsynch protocol, have successfully addressed the initial limitations by significantly improving the estrous response, ovulation, and pregnancy rates in buffaloes.

The Heatsynch protocol (Table 1), which is similar to Ovsynch but replaces the day 9 GnRH injection with estradiol (E2) on day 8, resulted in an 80% ovulatory response in Murrah buffaloes. Mirmahmoudi et al. developed the Estradoblesynch protocol, which involves the administration of PGF2α two days prior to the Heatsynch protocol. This protocol achieved pregnancy rates of 62% in cyclic Murrah buffaloes and 64% in anestrus Murrah buffaloes [41,42,43]. Based on a meta-analytical assessment considering 32 articles encompassing 4003 buffaloes, a comparison between the Ovsynch and modified Ovsynch protocols in water buffaloes revealed that buffaloes treated with the modified Ovsynch exhibited higher pregnancy per artificial insemination (P/AI) rates compared to those treated with the standard Ovsynch protocol. Cyclic buffaloes demonstrated higher P/AI rates compared to non-cyclic buffaloes across various estrus synchronization protocols. Although the Heatsynch protocol increases labor, the dramatic improvement in P/TAI after Heatsynch supports the use of this protocol for programming the reproductive management in water buffaloes [44]. While the modified protocols incorporating estradiol offer significant improvements in pregnancy per artificial insemination (P/AI) rates and reduce the need for estrus detection—a critical challenge in buffaloes—the increased costs and labor associated with these protocols must be carefully weighed as the cost per gestation can often be lower. Nonetheless, these protocols represent valuable alternatives in the reproductive management of water buffaloes, particularly for improving reproductive outcomes in cyclic animals.

#### 2.1.2. Prostaglandins

The use of prostaglandins (PGs) (Table 1) is a well-established method for ES in buffaloes. Administering prostaglandins induces luteolysis, lowering progesterone levels and leading to estrus within 50–96 h [45,46]. Effective synchronization requires cycling animals because PG induce luteolysis, which synchronizes the follicular phases by allowing the dominant follicle (DF) to mature, leading to estrus and ovulation. For synchronization, it is essential that the animals have a functional corpus luteum (CL) since cycling animals may lack PG-responsive CL, which are necessary for the synchronization process. The response to PG is influenced by the size and status of the DF and CL at administration [47,48]. High estrus rates are typical during the breeding season, with conception rates influenced by interval from calving, nutrition, and season. For example, estrous rates were 60% during the low breeding season (reduced fertility due to seasonality) with poor conception rates, but 86.6% during the breeding season with conception rates of 47.8–53% [49,50]. The intra-vulvo-submucosal approach has been dismissed because it proved to be poorly effective despite the lower dose, resulting in a higher cost per pregnancy compared to the intramuscular route [51]. Administering two PG injections 11–12 days apart during the breeding season has shown satisfactory results for cyclic animals in well-managed herds using natural service or timed AI (estrous rate 25–95% with conception rate 22.8–83%) [29,52]. Although the results are promising at reduced costs, the use of prostaglandins alone is not as popular as protocols that combine them with other substances, such as gonadorelins or progesterone. This is likely due to the additional procedures required, such as identifying cyclic animals and determining the estrous period when using prostaglandin exclusively.

#### 2.1.3. Progesterone

Progesterone synchronization protocols (Table 1) offer a reliable method to control and enhance reproductive performance in buffaloes. By providing a controlled and predictable interval to estrus and ovulation, progesterone-based protocols facilitate efficient breeding programs, particularly in large herds where estrus detection is challenging. Progesterone inhibits the suppression of both FSH and LH, which promotes the atresia of all follicles present on the ovary in cattle and buffaloes [53]. Once the dominant follicle (DF) undergoes atresia, its suppressive effects on other small follicles and FSH are removed, allowing a new follicular wave to begin. This leads to the formation of a new DF with ovulatory capacity following the withdrawal of the progesterone implant. The use of estradiol or GnRH at the time of implant insertion significantly suppresses DF growth and enhances the emergence of a new wave [54].

Despite the higher costs and labor requirements, the benefits in terms of improved fertility rates and efficient breeding processes make progesterone synchronization a valuable tool in buffalo reproduction management. Progesterone administration in buffaloes, through intravaginal devices (CIDR, PRID) or injections, offers controlled estrous regulation and improved reproductive efficiency; however, devices provide consistent hormone release but can be costly and require skilled handling, while injections are easier to administer but necessitate frequent handling and can cause stress to the animals [55]. In a progesterone-based protocol, improved ovulation synchronization compared to the Ovsynch protocol (78% vs. 50%) may be attributed to adequate progesterone priming of the hypothalamic system, which is essential for the optimal growth of the ovulatory follicle and the synchronized luteinizing hormone (LH) surge [56]. The implementation of progesterone-based protocols has been shown to markedly enhance conception rates, achieving a notable 60% success rate compared to the 30% observed with Ovsynch protocols. This substantial improvement underscores the efficacy of progesterone priming in optimizing reproductive outcomes [56,57]. These benefits contribute to enhanced fertility rates and more efficient reproductive management. However, a significant drawback of these protocols is the increased risk of microbial infections. The intravaginal devices can create a conducive environment for the growth of bacteria, leading to conditions such as vaginitis or endometritis. These infections can compromise animal health, leading to discomfort, reduced reproductive performance, and potential long-term consequences for fertility. The presence of the device may disrupt the natural vaginal flora and act as a reservoir for pathogens, further exacerbating the risk of infection.

**Table 1:** Estrous synchronization protocols currently used in buffaloes: characteristics of the protocols, estrous induction rate, and fertility performance.

Name of Synchronization Protocol	Protocol	Estrous Induction Rate	Fertility Rate	Bibliography
Ovsynch	Day 0 GnRH Day 7 PGF2α Day 9 GnRH Day 10 AI	60% of acyclic buffaloes 81% of cyclic buffaloes, 75.8%	20% 20–40%, 18%, 35.8%	[34,35,39]
Insulin Ovsynch protocol	Day 0 GnRH Day 7 PGF2α Day 9 Insulin Day 10 AI	81.9%	66.66%, 40%	[31,39]
Double Ovsynch protocol	Day 0 GnRH Day 7 PGF2α Day 9 GnRH Day 16 PGF2α Day 18 GnRH Day 19 AI	88.3%	44.4%	[38]
Mifepristone Ovsynch protocol	Day 0 GnRH Day 7 PGF2α Day 9 GnRH and mifepristone Day 10 AI	86.1%, 82.4%	45.4%	[40]
Progesterone protocols	Day 0–Day 15 PRID	78%	60%	[56,57]
Prostaglandins protocol	Day 0 PGF2α Day 11 PGF2α 50–96 h AI	60% breeding season 86.6% breeding season	47.8–53%	[49,50]
Heatsynch protocol	Day 0 GnRH Day 7 PGF2α Day 9 Estradiol Day 10 AI	80%	62%	[41]
Estradoublesynch protocol	Day 0 PGF2α Day 2 GnRH Day 9 PGF2α Day 11 Estradiol Day 12 AI	85%	64%	[42,43]

### 2.2. Artificial Insemination

Artificial insemination in buffaloes primarily utilizes the recto-vaginal technique, which is the most widely adopted method due to its practicality and effectiveness. This technique involves the insertion of an AI gun through the cervix into the uterus, guided by the palpation of the reproductive tract through the rectum. Clinically, AI is used to enhance genetic improvement, increase reproductive efficiency, and manage breeding schedules. It allows for the use of semen from genetically superior bulls, which may not be locally available, thereby accelerating genetic progress. Additionally, AI helps in controlling the spread of venereal diseases and enables the precise timing of insemination through synchronization protocols, ultimately improving conception rates and overall herd productivity [58,59]. Various strategies have been explored to detect the estrus of female buffalo. These strategies include the use of teaser bulls, radiotelemetry (100% accuracy), pedometers (75% accuracy), and the development of multiple estrus synchronization protocols [60,61]. Despite undergoing ES and AI, only about 40–50% of female buffalo conceive. However, by re-synchronizing and performing AI again on those that do not conceive, the conception rate can reach approximately 95% after three AI cycles over 93 days, with successful application in both pluriparous buffalo and buffalo heifers, incorporating ovarian status and pregnancy monitoring, and varying protocols based on the category of female and breeding season [62].

## 3. Hormonal Interventions and Protocols for Optimal Ovulation and Conception

Ovulation and CL organization in buffaloes are stimulated using various hormones to enhance reproductive efficiency and fertility. GnRH is administered to trigger the release of LH and FSH from the pituitary gland, leading to ovulation and subsequent formation of the CL. In buffalo, the administration of GnRH has proven effective in inducing ovulation in approximately 80% of females, thereby making it highly suitable for use in FTAI protocols [63]. The efficacy of TAI protocols is significantly influenced by the size of the ovarian follicle at the time of induction, as larger follicles result in the formation of larger corpora lutea. These larger corpora lutea secrete higher levels of progesterone, which is crucial for maintaining pregnancy and enhancing fertility in both buffaloes and cattle. The absolute concentrations of progesterone, particularly when they fall below a critical threshold during the first seven days post-ovulation, can significantly impact the establishment of pregnancy [64,65]. However, while cows generally have larger ovarian follicles compared to heifers, this does not always translate to improved reproductive outcomes, particularly in older animals where hepatic steroid clearance may interfere with the results. Buffaloes present unique challenges for AI due to their subtle estrus signs and delayed ovulation. Despite these challenges, there are effective strategies to enhance reproductive success, such as utilizing TAI during specific ovulation windows through protocols like Ovsynch- or progesterone-based approaches. Research has shown that ovulation in beef and buffalo cows typically occurs between 65 and 75 h after the removal of a CIDR (controlled internal drug release) device. Understanding this timing is crucial for optimizing reproductive outcomes. However, administering GnRH and performing TAI at 72 h post-CIDR removal in a 7-day CIDR Co-synch protocol may result in suboptimal oocyte development if smaller follicles are induced to ovulate [63,66]. This could negatively affect embryonic and fetal survival through the ability of the follicle to respond to the LH surge or to develop a corpus luteum capable of supporting the establishment of pregnancy. In contrast, buffalo heifers subjected to GnRH/TAI at either 72 or 84 h after CIDR removal demonstrated strong estrus intensity in the 7-day CIDR Co-synch protocol. This finding is significant because higher estrus intensity has been positively correlated with improved conception rates in conventional farming systems [67].

The luteinizing hormone (LH) directly induces ovulation and supports the development and function of the CL. It is critical for the final maturation of the ovarian follicle and the release of the oocyte. Human chorionic gonadotropin (hCG) mimics LH and is used to induce ovulation. It helps in the luteinization of the follicle in buffaloes [67], ensuring proper CL formation and function. Equine chorionic gonadotropin (eCG) has both LH and FSH activity, promoting follicular development and ovulation, as well as supporting CL formation. When utilizing the Ovsynch protocol for FTAI in buffaloes during the breeding season, the substitution of the final GnRH administration with human hCG (1500 UI) has been shown to increase plasma progesterone (P4) concentrations. However, this modification did not result in an improvement in conception rates [67]. Consequently, during periods characterized by reduced daylight length, either GnRH or hCG treatment could be effectively employed without significant differences in outcomes [68].

Even if progesterone therapy post-AI has not been extensively investigated as a method to improve reproductive characteristics in buffaloes, preliminary findings suggest its potential benefits. By enhancing the hormonal environment necessary for ovulation and providing an endometrial support for early embryo development, including interferon secretion and establishing of gestation, progesterone therapy may significantly boost conception rates and overall reproductive efficiency. Studies, such as those by Butani et al. (2016) [68], have demonstrated that the administration of progesterone therapy on the fourth or fifth day post-insemination can enhance conception rates by 10–20% in repeat breeding buffaloes. This therapeutic approach addresses the hormonal imbalances that often underlie repeat breeding issues. Additionally, the presence of mild genital infections, which are prevalent in repeat breeders, can be effectively managed with targeted antimicrobial treatments through either intrauterine infusions or parenteral administration of antibiotics [69]. This dual approach of hormonal therapy and infection control facilitates optimal corpus luteum function and increases the likelihood of successful conception and maintenance of pregnancy, thereby achieving the desired calving intervals under field conditions. These hormonal interventions are essential in reproductive management, enabling precise control over the timing of ovulation and improving the synchronization of estrus cycles, thereby enhancing the success rates of artificial insemination and overall reproductive performance in buffalo herds. 

## 4. Optimizing the Interval to AI and Fertility in Buffalo Herds: Diagnosis and Treatment of Common Functional and Inflammatory Pathologies Affecting the Reproductive System

Diagnosing and managing reproductive pathologies in buffaloes require a combination of clinical examination, advanced diagnostic tools like ultrasonography, and targeted therapeutic interventions. Hormonal treatments play a crucial role in addressing ovarian dysfunctions, while antibiotics and anti-inflammatory drugs are essential for managing infections and postpartum complications. Effective reproductive health management can significantly enhance fertility and productivity in buffalo herds. A few of the most common reproductive pathologies in buffaloes will be described and analyzed below in terms of diagnosis, treatment, and prognosis.

### 4.1. Anestrus

It is a significant reproductive disorder in dairy buffaloes, characterized by the absence of estrus behavior. A study conducted in southern Nepal [69] aimed to elucidate the causes of anestrus in buffaloes and evaluate their reproductive performance following treatment under field conditions. The study examined 104 anestrus buffalo cows and 111 heifers, revealing that 61.4% of cows and 76.6% of heifers experienced true anestrus with ovarian dysfunction, while 33.3% of cows and 18.9% of heifers exhibited silent ovulation. Notably, the anestrus duration exceeded six months in 83% of cows and ten months in 61.5% of cows. Additionally, 67.4% of both cows and heifers had anestrus intervals longer than five months from the last breeding to diagnosis. Treatment with PGF2α in cows and heifers possessing a CL resulted in significantly higher pregnancy rates within one to two months compared to vitamin/mineral mixtures [70]. Furthermore, the administration of gonadotropin-releasing hormone to cows and heifers with inactive ovaries and dominant follicles also significantly improved pregnancy rates within one month. These findings indicate that true anestrus with inactive ovaries is the predominant cause of anestrus in dairy buffaloes, with prolonged durations of anestrus post-calving and post-breeding. Routine reproductive examinations and appropriate hormonal treatments are recommended to enhance reproductive performance in affected buffaloes [71,72]. Recent studies have challenged the earlier literature by demonstrating that in true anestrus buffaloes, normal follicular recruitment and the emergence of follicular waves do occur. However, the DF ultimately fails to ovulate and regresses, likely due to disruptions during the critical phases of selection, deviation, and dominance [73,74]. El-Wishy (2007) [75] reviewed four main forms of anestrus: true anestrus (characterized by inactive ovaries and small to medium-sized anovulatory follicles), subestrus, prolonged luteal activity, and ovarian cysts, in addition to pregnancy. Differentiating between true anestrus and subestrus is particularly important in buffaloes due to their subtle estrous signs. However, the accuracy of a single rectal palpation of the ovaries is limited, often resulting in an overestimation of true anestrus due to the misdiagnosis of the corpus luteum. 

The induction of estrus and improvement in fertility rates in anestrus buffaloes have been effectively managed through therapeutic interventions using insulin and GnRH. A comparative study explored the efficacy of these treatments, providing a detailed numerical analysis. Buffaloes treated with insulin showed a remarkable estrus induction rate of 88.9%, with 24 out of 27 buffaloes displaying signs of estrus. Furthermore, the pregnancy rate in this group was 66.7%, with 18 out of 27 buffaloes successfully conceiving. In contrast, buffaloes treated with GnRH exhibited an estrus induction rate of 77.8%, with 21 out of 27 buffaloes coming into estrus, and a pregnancy rate of 55.6%, with 15 out of 27 buffaloes conceiving [73]. These numbers clearly highlight the superior efficacy of insulin over GnRH in managing anestrus. The role of insulin in this context is particularly noteworthy. Insulin stimulates the hypothalamic–pituitary–gonadal axis, enhancing the secretion of GnRH from the hypothalamus and subsequently increasing LH release from the pituitary gland. This hormonal cascade is crucial for promoting follicular development and ovulation. Additionally, insulin administration increases peripheral and intra-follicular levels of insulin-like growth factor, which plays a significant role in ovarian follicle growth and maturation. Despite insulin’s association with GnRH secretion at the hypothalamus, mice with knockout (KO) insulin receptors at the arcuate nucleus (ARC) exhibit normal fertility. In contrast, the same knockout with leptin receptors results in blocked reproductive axes and sterility in mice. Therefore, leptin, a key signal in the central regulation of energy homeostasis that ultimately modulates the reproductive axis, is as important as insulin, moving in the blood similarly. The study underscores the potential of insulin therapy not only in overcoming anestrus but also in significantly enhancing reproductive performance and productivity in buffaloes. This therapeutic approach offers a promising solution to address reproductive challenges in buffalo herds, thereby contributing to improved fertility management in agricultural practices [76,77].

Progesterone is commonly used in the treatment of anestrus in buffaloes, particularly through the administration of controlled internal drug release (CIDR) devices or progesterone-releasing intravaginal devices (PRIDs). These devices release progesterone over a period, which mimics the luteal phase of the estrous cycle. After a set period, typically 7 to 10 days, the device is removed, and this withdrawal mimics the natural decline of progesterone, leading to the resumption of estrous cycles and ovulation. This treatment is often combined with the administration of prostaglandin F2α (PGF2α) or equine chorionic gonadotropin (eCG) to enhance the synchronization of estrus and improve conception rates [78,79]. Estrus induction rates in anestrous buffaloes following progesterone treatment typically range from 60% to 80%. Pregnancy rates after progesterone-induced estrus can vary widely, generally falling between 30% and 50%, depending on the protocol used and the season. Success rates tend to be higher during the breeding season due to the natural enhancement in reproductive activity [74,78]. 

### 4.2. Ovarian Cysts

Ovarian cysts represent a significant reproductive pathology in buffaloes, with an incidence rate varying widely from 3% to 30% based on different studies and geographical locations [80]. The etiology of ovarian cysts in buffaloes is multifactorial, involving endocrine imbalances, particularly disruptions in the hypothalamic–pituitary–ovarian axis. This disruption leads to an altered secretion of gonadotropins, resulting in the failure of the DF to ovulate [80,81]. Factors such as nutritional deficiencies, stress, and genetic predisposition contribute to the development of ovarian cysts. Research on the endocrine profiles of cows with cystic ovarian disease (COD) has shown varied serum LH concentrations—either elevated or normal—while serum FSH levels are typically low or normal. Serum inhibin levels are generally high, and there is often a deficiency in LH and FSH receptors at the ovarian level [81].

The management of ovarian cysts in buffaloes has employed various hormonal therapies with notable success. GnRH treatment, in particular, has proven effective in inducing luteinization and ovulation of cystic follicles, thereby resolving the cystic condition. Specifically, 70% of buffaloes treated with GnRH exhibited a reduction in cyst size, and 60% resumed normal estrous cycles within 21 days post-treatment [82]. The combination of GnRH and PGF2α has proven effective for treating ovarian cysts, achieving a 65% resolution rate. Progesterone therapy has also been beneficial, with a 55% success rate in facilitating cyst regression and restoring normal ovarian function [83].

Echotexture analysis provides valuable insights into the internal structure of ovarian cysts, which helps predict the success of various treatments. For example, buffaloes with cysts exhibiting a homogeneous echotexture responded well to GnRH treatment, with a 70% reduction in cyst size and 60% resuming normal estrous cycles within 21 days. In contrast, cysts with a heterogeneous echotexture had less favorable outcomes, with only a 45% resolution rate. This differentiation is crucial, as it allows veterinarians to tailor treatments based on the cyst’s echotexture, optimizing therapeutic efficacy [82].

The GnRH and PGF2α combination, for instance, achieved a 65% success rate in resolving cysts with a more complex echotexture, demonstrating its versatility. Echotexture analysis enhances the precision of ovarian cyst treatment in buffaloes by ensuring that therapeutic strategies are appropriately targeted [83,84].

Follicular cysts, which have thin walls and anechoic fluid content, were effectively treated with GnRH, resulting in a 75% resolution rate and a subsequent resumption of normal estrous cycles in 65% of cases. Luteal cysts, characterized by thicker walls and mixed echogenic content, responded well to PGF2α treatment, with a 70% resolution rate. The combined GnRH/PGF2α protocol achieved an 80% success rate in resolving cysts with mixed echotexture. This echotexture-guided methodology not only improves diagnostic accuracy but also ensures that buffaloes receive the most effective therapy tailored to the specific nature of their ovarian cysts [80,82].

### 4.3. Metritis and Endometritis

Metritis and endometritis are significant reproductive disorders affecting buffaloes postpartum, often leading to reduced fertility and economic losses in herds. Typically, metritis is characterized by inflammation of the uterine lining shortly after calving, often due to bacterial infections introduced during parturition. Endometritis, on the other hand, involves prolonged inflammation of the endometrium, which can persist if not adequately treated [85].

Postpartum metritis is a significant reproductive challenge in buffalo cows, often triggered by retained placenta and puerperal pathology. Studies have identified Escherichia coli, Archanobacterium pyogenes, Bacteroides fragilis, and Fusobacterium necrophorum as the predominant bacteria in uterine infections affecting buffaloes [85]. Among these, A. pyogenes and F. necrophorum are particularly implicated in severe uterine inflammation, as confirmed by bacteriological examinations. Treatment strategies for postpartum metritis in buffaloes typically involve systemic administration of oxytetracycline combined with PGF2α. This method has demonstrated effective clinical cure rates, significantly enhancing reproductive health outcomes in these animals. Notably, intrauterine infusion of oxytetracycline did not offer additional benefits compared to systemic administration for treating uterine infections [86]. However, PGF2α has been observed to improve clinical recovery in buffaloes with postpartum metritis, highlighting its crucial role in managing this condition. Understanding these treatment dynamics is essential for implementing targeted therapeutic interventions to mitigate postpartum uterine infections in buffalo herds and protect their reproductive performance [87,88].

Endometritis is a specific disease that can cause infertility. Retained placenta was identified as a predisposing factor in 13.3% of cases. Bacterial analysis revealed *Escherichia coli* in 23% of uterine samples, *Archanobacterium pyogenes* in 13%, and Staphylococcus aureus in 10%. Diagnostic methods combining vaginoscopy and transrectal palpation, along with cytogenic examination of uterine discharge, showed significant correlations between vaginal mucus character scores and bacterial growth density scores. Polymorphonuclear cell (PMN) counts were notably higher (*p* < 0.01) in buffaloes with repeat breeding compared to the controls, particularly in cases infected with A. pyogenes. Elevated creatine kinase (CK) levels (321.47 ± 39.06 U/L and aspartate aminotransferase (AST) activities (133.93 ± 12.43 U/L) underscored the severity of inflammation. Treatment with oxytetracycline and tylosin, either locally or systemically, resulted in clinical cure and improved pregnancy outcomes across all treatment groups, although estradiol supplementation did not influence cure rates or pregnancy success. These findings underscore the efficacy of targeted antibiotic therapy in managing endometritis and highlight the importance of accurate diagnostic protocols in buffalo reproductive health management [85,88]. The bacteriological analysis of the uterine environment revealed that Arc. pyogenes exhibited high sensitivity to amoxicillin/clavulanic acid (97.3%), bacitracin (96.7%), ceftiofur (95.8%), and cephapirin (77.5%). *E. coli* exhibited the highest sensitivity to norfloxacin (98.1%), marbofloxacin (95.8%), gentamycin (88%), amoxicillin/clavulanic acid (80.7%), and ceftiofur (73.1%). Resistance rates were notable for oxytetracycline, with Arc. pyogenes and *E. coli* showing resistance levels of 63.7% and 31%, respectively [86].

In a study on the treatment of puerperal metritis in buffaloes [89], a combination therapy involving intrauterine infusion of 30 mL of 2% povidone-iodine and systemic administration of cefoperazone at a dose of 10 mg/kg body weight for 3 days, along with a single intramuscular injection of 500 µg cloprostenol, was evaluated. Out of 18 buffaloes treated, 17 (94.44%) showed significant clinical improvement, with clear, non-fetid uterine secretions observed 14 days post-treatment and during subsequent estrus. Of these, nine buffaloes (52.9%) conceived on the first artificial insemination (AI), six (35.3%) conceived on the second AI, and one buffalo conceived after the third AI. These results underscore the effectiveness of the treatment regimen in achieving clinical cure and promoting uterine involution, though further studies on larger populations are recommended to confirm these findings [89]. A case of clinical endometritis in a buffalo was diagnosed based on hemato-biochemical analysis, which revealed monocytosis, slightly elevated AST, hyperglobulinemia, and hypoalbuminemia, along with clinical examination and laboratory findings. Initial treatment focused on managing pyrexia, while the actual treatment commenced after the antibiotic sensitivity testing (ABST) results. The buffalo was treated with flunixin meglumine at 1.1 mg/kg intramuscularly twice a day for 3 days as an anti-inflammatory, antipyretic, and analgesic. Additionally, amikacin sulfate was administered at 22 mg/kg intramuscularly once daily for 5 days to address the infection. Supportive therapy included a single intramuscular injection of Ferritas (1 mL/10 kg) as an iron supplement, along with Inj. Avil (15 mL I/M) and Inj. Toxol (20 mL I/M) for 5 days. An intrauterine wash was performed using 0.9% NaCl, with continuous flushing to irrigate mucopurulent discharge, followed by a final lavage with 20 mL of amikacin. The buffalo showed signs of improvement the following day and was fully recovered by the sixth day post-treatment, confirmed by a negative result on the vaginal mucus white side test [90]. Despite the effectiveness of these treatments, future research on metritis and endometritis therapy should focus on natural approaches that do not interfere with milk production and, consequently, economic indicators.

### 4.4. Retention of the Placenta

Retention of the placenta is a significant pathological issue encountered by farmers and field veterinarians. This condition incurs substantial financial losses due to the costs associated with medical treatments, delays in uterine involution, extended intervals before the first postpartum estrus, decreased fertility rates, and prolonged calving intervals. Typically, the placenta is expelled within 0.5 to 8 h after parturition in cattle and buffaloes [91]. The incidence of retention of the placenta in buffaloes ranges from 10–15%, with slightly higher values (16–18%) reported in some studies [92], and it increases with parity, twins, and premature births, varying by country, year, and herd. The normal time for placenta expulsion averages 3.33 h, with similar values (4.56–4.93 h) reported in buffaloes [93,94]. 

In cases of retained placenta in buffaloes, manual removal followed by comprehensive antibiotic therapy is a common practice. According to studies, manual removal was performed in a majority of cases (69.56%) [95,96], with subsequent administration of oxytocin, calcium, and antibiotics, both locally and systemically. Specific treatments included intrauterine infusion of oxytetracycline solution (40 mL daily for three days), resulting in the resolution of unhealthy discharges within 3–5 days in all treated animals [97]. Successful recovery and resumption of milk production were observed subsequently. Furthermore, systemic administration of antibiotics such as strepto penicillin, meloxicam, and chlorpheniramine maleate, coupled with intramuscular and intravenous infusions of fluids and vitamins, contributed to the effective management of retained placenta and associated complications. These therapeutic approaches highlight the importance of timely intervention and comprehensive management strategies in improving the health outcomes of buffaloes affected by retained placenta [97]. In any case, the therapeutic management of retained placenta has been relatively understudied in recent times. New studies focusing on the development of natural alternatives for the effective and healthy treatment of retained placenta in buffaloes are essential. Solutions incorporating substances such as mineral rocks, purgatives, astringents, and other natural compounds could offer a promising approach. These mixtures, when carefully formulated, may help resolve retained placenta without the need for antibiotics, thus minimizing the risk of antibiotic residues in milk and reducing the potential impact on both animal health and economic indicators.

### 4.5. Repeat Breeding

Repeat breeding in buffaloes refers to the condition where a buffalo fails to conceive after three or more consecutive artificial inseminations or natural services, despite showing regular estrous cycles. This condition is associated with various factors such as poor semen quality, improper timing of insemination, uterine infections, or suboptimal nutrition [98]. Repeat breeding in buffaloes is characterized by a varied incidence ranging from 0.70% to 30% across different studies, largely influenced by factors such as seasonal fertility suppression, particularly notable during spring and winter calving periods [99]. First parity, peri-parturient diseases, and lactation are significant predisposing factors. Etiologies include failure of fertilization and early embryonic deaths, with identified causes of fertilization failure being limited in buffaloes [100]. Ovulatory disturbances and ovarian cysts are infrequent, often with subtle clinical signs. Endometritis emerges as a primary female factor contributing to fertilization failures, while poor semen quality and improper insemination practices are key male factors [101]. Early embryonic deaths are prevalent among buffaloes inseminated late in the breeding season, associated with low luteal progesterone levels. Diagnostic strategies involve vaginoscopic and transrectal examinations, uterine cytology for assessing genital health, and advanced techniques like ultrasonography and hysteroscopy for detailed evaluation of ovarian and uterine functions, although definitive diagnosis remains challenging [102].

The economic impact of repeat breeding in dairy buffalo is profound, affecting both milk production and reproductive efficiency. Economic losses arise from extended dry periods, reduced calving frequency, and diminished lifetime productivity due to delayed conception and increased calving intervals [100]. These losses are exacerbated by conditions such as sub-fertility, infertility, and sterility, leading to higher management costs and necessitating the premature culling of affected animals. Factors contributing to repeat breeding include seasonal influences, peri-parturient disorders, and metabolic disturbances, all of which impair reproductive health and productivity in buffalo herds. Effective management strategies involving accurate heat detection, embryo transfer techniques, and hormonal treatments at insemination are crucial in mitigating these economic losses and improving overall herd fertility [101,102].

In reproductive management strategies for buffaloes, the administration of GnRH at insemination has demonstrated significant benefits for improving conception rates in repeat breeder cows. Studies indicate that GnRH treatment can increase conception rates by as much as 7.6% to 25% [103,104]. Similarly, the use of human chorionic gonadotropin (hCG) following artificial insemination has been studied extensively. Research by Santos et al., 2001, [105] found that hCG administered on day 5 post-insemination resulted in a substantial increase in conception rates. Specifically, hCG treatment led to a higher percentage of cows with more than one corpus luteum (86.2% vs. 23.2% in controls), increased plasma progesterone concentrations, and improved conception rates on day 28 (45.8% vs. 38.7%), day 45 (40.4% vs. 36.3%), and day 90 (38.4% vs. 31.9%) compared to untreated females.

Furthermore, integrating both artificial insemination (AI) and natural service (NS) in reproductive management programs has proven beneficial in reducing repeat breeding incidences. Dairy farms adopting a combined approach have observed improved fertilization rates compared to those relying only one method [106]. Effective management of service bulls in AI and NS programs is crucial to optimize breeding outcomes and minimize fertility issues.

Additionally, re-synchronization protocols play a pivotal role in enhancing reproductive efficiency in dairy cows. More than 50% of lactating cows fail to conceive after their first AI, often due to inadequate estrus detection and timing of insemination. Implementing re-synchronization protocols involves administering GnRH at non-pregnancy diagnosis followed by re-insemination approximately 10 days later, effectively reducing the interval between inseminations and improving pregnancy rates [105]. These strategies underscore the importance of precise timing and comprehensive management practices in mitigating repeat breeding and optimizing reproductive performance in dairy herds.

## 5. Conclusions

Among biotechnologies in animal reproduction, AI is the most widely used method for buffaloes. Due to specific management systems, AI often requires estrus synchronization, typically using the Ovsynch protocol or its various modifications, including the incorporation of insulin in place of GnRH. Key challenges such as anestrus and repeat breeding syndromes remain prevalent, highlighting the need for continued research. Practitioners should aim to reduce reliance on hormonal and antibiotic treatments by exploring natural alternatives to enhance fertility and shorten the interval to successful AI.

## Data Availability

Not applicable.
